# *N*-(3,5-Dimeth­oxy­phen­yl)benzamide

**DOI:** 10.1107/S1600536811019350

**Published:** 2011-06-11

**Authors:** Hong-Lei Li, Jiang-Tao Cui

**Affiliations:** a Institute of Functional Biomolecules, State Key Laboratory of Pharmaceutical Biotechnology, Nanjing University, Nanjing 210093, People’s Republic of China

## Abstract

The title compound, C_15_H_15_NO_3_, was prepared by stirring benzoyl chloride with 3,5-dimeth­oxy­aniline in dioxane at ambient temperature. The dimeth­oxy­phen­yl–amide segment of the mol­ecule is almost planar, with a C—N—C=O torsion angle of −4.1 (4)°. The two benzene rings are inclined at an angle of 76.66 (13)°. In the crystal, inter­molecular N—H⋯O inter­actions generate centrosymmetric dimers..

## Related literature

For related structures, see: Faler & Joullie (2006 ▶); Hadjeri *et al.* (2002 ▶); Beney *et al.* (2000 ▶). For bond lengths and angles in related structures, see: Saeed *et al.* (2010 ▶); Wang *et al.* (2010 ▶); Anderson *et al.* (2005 ▶).
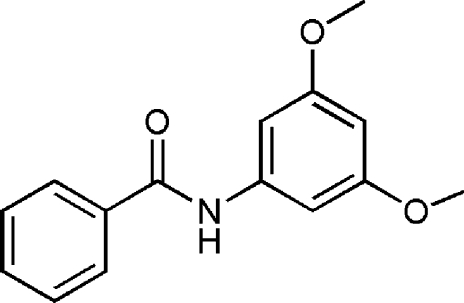


## Experimental

### 

#### Crystal data

C_15_H_15_NO_3_
*M* *_r_* = 257.28
Monoclinic, 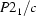

*a* = 8.0390 (16) Å
*b* = 20.003 (4) Å
*c* = 9.2710 (19) Å
β = 111.39 (3)°
*V* = 1388.1 (5) Å^3^
*Z* = 4
Mo *K*α radiation
μ = 0.09 mm^−1^
*T* = 293 K
0.30 × 0.30 × 0.10 mm

#### Data collection

Enraf–Nonius CAD-4 diffractometer
Absorption correction: ψ scan (North *et al.*, 1968) ▶ *T*_min_ = 0.975, *T*_max_ = 0.991
2737 measured reflections
2550 independent reflections
1564 reflections with *I* > 2σ(*I*)
*R*_int_ = 0.033
3 standard reflections every 200 reflections  intensity decay: 1%

#### Refinement

*R*[*F* ^2^ > 2σ(*F* ^2^)] = 0.054
*wR*(*F* ^2^) = 0.165
*S* = 1.00
2550 reflections
173 parameters
H-atom parameters constrained
Δρ_max_ = 0.17 e Å^−3^
Δρ_min_ = −0.19 e Å^−3^

### 

Data collection: *CAD-4 EXPRESS* (Enraf–Nonius, 1994 ▶); cell refinement: *CAD-4 EXPRESS*; data reduction: *XCAD4* (Harms & Wocadlo, 1995 ▶); program(s) used to solve structure: *SHELXS97* (Sheldrick, 2008 ▶); program(s) used to refine structure: *SHELXL97* (Sheldrick, 2008 ▶); molecular graphics: *SHELXTL* (Sheldrick, 2008 ▶); software used to prepare material for publication: *publCIF* (Westrip, 2010 ▶).

## Supplementary Material

Crystal structure: contains datablock(s) global, I. DOI: 10.1107/S1600536811019350/zj2010sup1.cif

Structure factors: contains datablock(s) I. DOI: 10.1107/S1600536811019350/zj2010Isup2.hkl

Supplementary material file. DOI: 10.1107/S1600536811019350/zj2010Isup3.cml

Additional supplementary materials:  crystallographic information; 3D view; checkCIF report

## Figures and Tables

**Table 1:** Hydrogen-bond geometry (Å, °)

*D*—H⋯*A*	*D*—H	H⋯*A*	*D*⋯*A*	*D*—H⋯*A*
N—H0*A*⋯O3^i^	0.86	2.14	2.831 (3)	137

Symmetry code: (i) 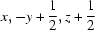.

**Fig. 1.**
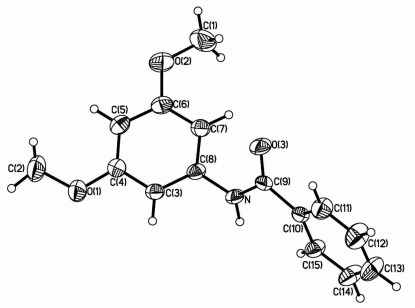
The structure of the title compound at 50% probability level.

**Fig. 2.**
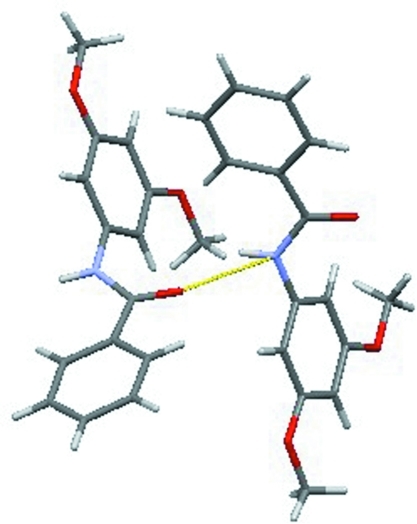
N···O contacts in title compound (yellow line) linking the molecules into centrosymmetric dimers.

**Fig. 3.**
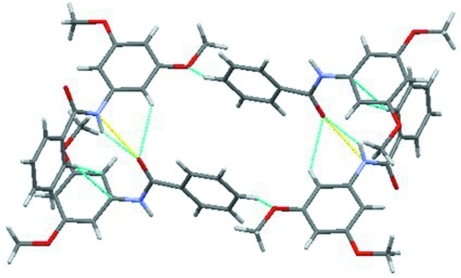
Crystal packing of title compound viewed down the b axis, with hydrogen bonds drawn as yellow lines and representative C–H interactions shown as light blue lines.

## supplementary crystallographic information

### Comment

In this paper we report the structural information for the title compound, C_15_H_15_NO_3_, obtained in our search for a strong anti-tumor reagent, for which the methoxyphenyl amide segment of the molecule is planar with a C8—N1—C9—O3 torsion angle of -4.1 (4)°. The two benzene rings are inclined at an angle of 76.66 (13)°. In the crystal structure, intermolecular O3···N interactions of 2.831 (3) Å, generate centrosymmetric dimmers, Fig 2. The packing is shown in Fig. 3. The bond lengths and angles of the title compound are in normal ranges when comparing with similar structures reported previously (Saeed *et al.* 2010; Wang *et al.* 2010; Anderson *et al.* 2005).

### Experimental

To a solution of 3, 5-dimethoxyaniline (1 mmol) in dry dioxane (2 ml) was added Et_3_N (1 mmol). The solution was stirred at ambient temperature for 10 min and treated by dropwise addition of benzoyl chloride (1 mmol). The reaction mixture was stirred at room temperature for 1 h then hydrolyzed by adding H_2_O and extracted with EtOAc. The organic layer was washed with brine, dried over anhydrous Na_2_SO_4_ and evaporated. The residue was purified by column chromatography eluted with petroleum ether: acetic ether (5:1) to N-(3, 5-dimethoxyphenyl) Benzamide as a light yellow powder and Yield 90%. Crystallization of the residue from methanol afforded the title compound (87%) as colourless crystals: ESI-MS TOF. calcd. for [*M*+Na]^+^: 280.09441; found: 280.09443.

### Refinement

The H atom on N1 was located in a difference Fourier map and refined isotropically. All other H-atoms were placed in calculated positions and refined using a riding model with d(C—H) = 0.93 Å, *Uiso* = 1.2*Ueq (C)* for aromatic and 0.96 Å, *Uiso* = 1.5*Ueq (C)* for the CH_3_ H atoms. The crystal was relatively weakly diffracting reducing the overall fraction of measured reflections.

### Crystal data

**Table:**

C_15_H_15_NO_3_	*F*(000) = 544
*M**_r_* = 257.28	*D*_x_ = 1.231 Mg m^−^^3^
Monoclinic, *P*2_1_/*c*	Mo *K*α radiation, λ = 0.71073 Å
Hall symbol: -P 2ybc	Cell parameters from 25 reflections
*a* = 8.0390 (16) Å	θ = 9–13°
*b* = 20.003 (4) Å	µ = 0.09 mm^−^^1^
*c* = 9.2710 (19) Å	*T* = 293 K
β = 111.39 (3)°	Block, colourless
*V* = 1388.1 (5) Å^3^	0.30 × 0.30 × 0.10 mm
*Z* = 4	

### Data collection

**Table:**

Enraf–Nonius CAD-4 diffractometer	1564 reflections with *I* > 2σ(*I*)
Radiation source: fine-focus sealed tube	*R*_int_ = 0.033
graphite	θ_max_ = 25.4°, θ_min_ = 2.0°
ω/2θ scans	*h* = 0→9
Absorption correction: ψ scan (North *et al.*, 1968)	*k* = 0→24
*T*_min_ = 0.975, *T*_max_ = 0.991	*l* = −11→10
2737 measured reflections	3 standard reflections every 200 reflections
2550 independent reflections	intensity decay: 1%

### Refinement

**Table:**

Refinement on *F*^2^	Secondary atom site location: difference Fourier map
Least-squares matrix: full	Hydrogen site location: inferred from neighbouring sites
*R*[*F*^2^ > 2σ(*F*^2^)] = 0.054	H-atom parameters constrained
*wR*(*F*^2^) = 0.165	*w* = 1/[σ^2^(*F*_o_^2^) + (0.093*P*)^2^] where *P* = (*F*_o_^2^ + 2*F*_c_^2^)/3
*S* = 1.00	(Δ/σ)_max_ < 0.001
2550 reflections	Δρ_max_ = 0.17 e Å^−^^3^
173 parameters	Δρ_min_ = −0.19 e Å^−^^3^
0 restraints	Extinction correction: *SHELXL97* (Sheldrick, 2008), Fc^*^=kFc[1+0.001xFc^2^λ^3^/sin(2θ)]^-1/4^
Primary atom site location: structure-invariant direct methods	Extinction coefficient: 0.037 (5)

### Special details

**Table:**

Geometry. All e.s.d.'s (except the e.s.d. in the dihedral angle between two l.s. planes) are estimated using the full covariance matrix. The cell e.s.d.'s are taken into account individually in the estimation of e.s.d.'s in distances, angles and torsion angles; correlations between e.s.d.'s in cell parameters are only used when they are defined by crystal symmetry. An approximate (isotropic) treatment of cell e.s.d.'s is used for estimating e.s.d.'s involving l.s. planes.
Refinement. Refinement of *F^2^* against ALL reflections. The weighted *R*-factor *wR* and goodness of fit *S* are based on *F^2^*, conventional *R*-factors *R* are based on *F*, with *F* set to zero for negative *F^2^*. The threshold expression of *F^2^*>σ(*F^2^*) is used only for calculating *R*-factors(gt) *etc*. and is not relevant to the choice of reflections for refinement. *R*-factors based on *F^2^*are statistically about twice as large as those based on *F*, and *R*-factors based on ALL data will be even larger.

### Fractional atomic coordinates and isotropic or equivalent isotropic displacement parameters (Å^2^)

**Table:**

	*x*	*y*	*z*	*U*_iso_*/*U*_eq_	
N	0.3527 (3)	0.25056 (10)	0.78089 (19)	0.0513 (5)	
H0A	0.3555	0.2581	0.8731	0.062*	
O1	0.4214 (3)	0.01660 (9)	0.8098 (2)	0.0754 (6)	
C1	0.8603 (5)	0.20349 (18)	0.5865 (5)	0.1074 (13)	
H1A	0.9541	0.1959	0.5476	0.161*	
H1B	0.9054	0.2297	0.6795	0.161*	
H1C	0.7641	0.2271	0.5102	0.161*	
O2	0.7975 (3)	0.14175 (11)	0.6190 (3)	0.0872 (7)	
C2	0.5014 (5)	−0.04432 (14)	0.7878 (4)	0.0954 (11)	
H2A	0.4455	−0.0813	0.8181	0.143*	
H2B	0.6266	−0.0438	0.8498	0.143*	
H2C	0.4859	−0.0488	0.6805	0.143*	
O3	0.2596 (3)	0.28887 (9)	0.53489 (18)	0.0732 (6)	
C3	0.3954 (3)	0.13183 (12)	0.7943 (2)	0.0505 (6)	
H3A	0.3059	0.1283	0.8351	0.061*	
C4	0.4817 (3)	0.07509 (12)	0.7710 (3)	0.0532 (6)	
C5	0.6135 (3)	0.07981 (13)	0.7111 (3)	0.0576 (7)	
H5A	0.6695	0.0415	0.6944	0.069*	
C6	0.6628 (3)	0.14226 (14)	0.6757 (3)	0.0578 (7)	
C7	0.5794 (3)	0.19976 (12)	0.6982 (2)	0.0533 (6)	
H7A	0.6134	0.2416	0.6751	0.064*	
C8	0.4436 (3)	0.19328 (12)	0.7564 (2)	0.0475 (6)	
C9	0.2622 (3)	0.29379 (11)	0.6675 (2)	0.0472 (6)	
C10	0.1626 (3)	0.34835 (11)	0.7090 (2)	0.0456 (6)	
C11	0.0282 (4)	0.38045 (14)	0.5912 (3)	0.0674 (8)	
H11A	−0.0003	0.3667	0.4892	0.081*	
C12	−0.0632 (4)	0.43259 (16)	0.6242 (4)	0.0859 (10)	
H12A	−0.1556	0.4531	0.5446	0.103*	
C13	−0.0196 (4)	0.45452 (16)	0.7731 (3)	0.0824 (9)	
H13A	−0.0789	0.4909	0.7943	0.099*	
C14	0.1106 (4)	0.42291 (15)	0.8899 (3)	0.0762 (9)	
H14A	0.1382	0.4371	0.9915	0.091*	
C15	0.2022 (3)	0.37003 (12)	0.8593 (3)	0.0561 (7)	
H15A	0.2913	0.3488	0.9403	0.067*	

### Atomic displacement parameters (Å^2^)

**Table:**

	*U*^11^	*U*^22^	*U*^33^	*U*^12^	*U*^13^	*U*^23^
N	0.0740 (13)	0.0518 (11)	0.0370 (9)	0.0088 (10)	0.0309 (9)	0.0003 (8)
O1	0.0848 (14)	0.0517 (11)	0.1028 (15)	0.0087 (10)	0.0497 (12)	0.0051 (10)
C1	0.093 (2)	0.108 (3)	0.152 (3)	−0.001 (2)	0.083 (2)	0.020 (2)
O2	0.0725 (13)	0.0954 (16)	0.1176 (18)	0.0032 (11)	0.0631 (13)	−0.0041 (13)
C2	0.106 (2)	0.0526 (18)	0.139 (3)	0.0147 (18)	0.059 (2)	−0.0014 (18)
O3	0.1181 (16)	0.0728 (13)	0.0434 (10)	0.0226 (11)	0.0468 (10)	0.0080 (8)
C3	0.0572 (14)	0.0549 (14)	0.0460 (13)	0.0036 (12)	0.0265 (11)	0.0021 (11)
C4	0.0544 (15)	0.0516 (15)	0.0547 (14)	0.0013 (12)	0.0212 (12)	0.0005 (11)
C5	0.0507 (14)	0.0616 (16)	0.0602 (15)	0.0077 (12)	0.0200 (12)	−0.0068 (12)
C6	0.0459 (14)	0.0732 (19)	0.0578 (15)	0.0014 (13)	0.0230 (12)	−0.0059 (13)
C7	0.0548 (15)	0.0582 (15)	0.0505 (13)	−0.0068 (12)	0.0233 (12)	−0.0044 (11)
C8	0.0543 (14)	0.0542 (14)	0.0359 (11)	0.0045 (11)	0.0186 (10)	−0.0026 (10)
C9	0.0627 (15)	0.0464 (13)	0.0406 (12)	−0.0052 (11)	0.0287 (11)	0.0011 (10)
C10	0.0527 (13)	0.0470 (13)	0.0419 (12)	−0.0042 (11)	0.0231 (11)	0.0015 (10)
C11	0.0783 (19)	0.0716 (17)	0.0452 (14)	0.0092 (15)	0.0138 (13)	−0.0006 (12)
C12	0.080 (2)	0.089 (2)	0.073 (2)	0.0328 (18)	0.0089 (16)	0.0047 (17)
C13	0.081 (2)	0.082 (2)	0.080 (2)	0.0323 (18)	0.0247 (17)	−0.0104 (16)
C14	0.083 (2)	0.090 (2)	0.0554 (16)	0.0262 (18)	0.0246 (15)	−0.0113 (15)
C15	0.0623 (16)	0.0633 (15)	0.0430 (13)	0.0122 (13)	0.0195 (12)	−0.0017 (11)

### Geometric parameters (Å, °)

**Table:**

N—C9	1.350 (3)	C5—C6	1.386 (4)
N—C8	1.421 (3)	C5—H5A	0.9300
N—H0A	0.8600	C6—C7	1.385 (3)
O1—C4	1.364 (3)	C7—C8	1.389 (3)
O1—C2	1.427 (3)	C7—H7A	0.9300
C1—O2	1.408 (4)	C9—C10	1.485 (3)
C1—H1A	0.9600	C10—C15	1.381 (3)
C1—H1B	0.9600	C10—C11	1.382 (4)
C1—H1C	0.9600	C11—C12	1.374 (4)
O2—C6	1.365 (3)	C11—H11A	0.9300
C2—H2A	0.9600	C12—C13	1.367 (4)
C2—H2B	0.9600	C12—H12A	0.9300
C2—H2C	0.9600	C13—C14	1.357 (4)
O3—C9	1.226 (2)	C13—H13A	0.9300
C3—C8	1.372 (3)	C14—C15	1.376 (3)
C3—C4	1.387 (3)	C14—H14A	0.9300
C3—H3A	0.9300	C15—H15A	0.9300
C4—C5	1.367 (3)		
			
C9—N—C8	123.69 (17)	C7—C6—C5	121.1 (2)
C9—N—H0A	118.2	C6—C7—C8	118.3 (2)
C8—N—H0A	118.2	C6—C7—H7A	120.9
C4—O1—C2	118.2 (2)	C8—C7—H7A	120.9
O2—C1—H1A	109.5	C3—C8—C7	121.2 (2)
O2—C1—H1B	109.5	C3—C8—N	118.17 (19)
H1A—C1—H1B	109.5	C7—C8—N	120.6 (2)
O2—C1—H1C	109.5	O3—C9—N	122.4 (2)
H1A—C1—H1C	109.5	O3—C9—C10	120.3 (2)
H1B—C1—H1C	109.5	N—C9—C10	117.24 (18)
C6—O2—C1	118.2 (2)	C15—C10—C11	118.5 (2)
O1—C2—H2A	109.5	C15—C10—C9	123.0 (2)
O1—C2—H2B	109.5	C11—C10—C9	118.5 (2)
H2A—C2—H2B	109.5	C12—C11—C10	120.3 (2)
O1—C2—H2C	109.5	C12—C11—H11A	119.9
H2A—C2—H2C	109.5	C10—C11—H11A	119.9
H2B—C2—H2C	109.5	C13—C12—C11	120.5 (3)
C8—C3—C4	119.3 (2)	C13—C12—H12A	119.7
C8—C3—H3A	120.3	C11—C12—H12A	119.7
C4—C3—H3A	120.3	C14—C13—C12	119.7 (3)
O1—C4—C5	124.7 (2)	C14—C13—H13A	120.2
O1—C4—C3	114.5 (2)	C12—C13—H13A	120.2
C5—C4—C3	120.8 (2)	C13—C14—C15	120.6 (2)
C4—C5—C6	119.3 (2)	C13—C14—H14A	119.7
C4—C5—H5A	120.3	C15—C14—H14A	119.7
C6—C5—H5A	120.3	C14—C15—C10	120.3 (2)
O2—C6—C7	124.0 (2)	C14—C15—H15A	119.8
O2—C6—C5	114.8 (2)	C10—C15—H15A	119.8
			
C2—O1—C4—C5	0.4 (4)	C9—N—C8—C3	−122.0 (2)
C2—O1—C4—C3	179.3 (2)	C9—N—C8—C7	59.1 (3)
C8—C3—C4—O1	−178.9 (2)	C8—N—C9—O3	−4.1 (4)
C8—C3—C4—C5	0.1 (3)	C8—N—C9—C10	175.41 (19)
O1—C4—C5—C6	179.8 (2)	O3—C9—C10—C15	−158.9 (2)
C3—C4—C5—C6	0.9 (4)	N—C9—C10—C15	21.6 (3)
C1—O2—C6—C7	2.3 (4)	O3—C9—C10—C11	18.8 (3)
C1—O2—C6—C5	−177.3 (3)	N—C9—C10—C11	−160.8 (2)
C4—C5—C6—O2	179.0 (2)	C15—C10—C11—C12	−0.2 (4)
C4—C5—C6—C7	−0.7 (4)	C9—C10—C11—C12	−177.9 (3)
O2—C6—C7—C8	179.9 (2)	C10—C11—C12—C13	1.7 (5)
C5—C6—C7—C8	−0.5 (3)	C11—C12—C13—C14	−2.5 (5)
C4—C3—C8—C7	−1.3 (3)	C12—C13—C14—C15	1.6 (5)
C4—C3—C8—N	179.8 (2)	C13—C14—C15—C10	−0.1 (4)
C6—C7—C8—C3	1.5 (3)	C11—C10—C15—C14	−0.7 (4)
C6—C7—C8—N	−179.6 (2)	C9—C10—C15—C14	177.0 (2)

### Hydrogen-bond geometry (Å, °)

**Table:**

*D*—H···*A*	*D*—H	H···*A*	*D*···*A*	*D*—H···*A*
N—H0A···O3^i^	0.86	2.14	2.831 (3)	137

Symmetry codes: (i) *x*, −*y*+1/2, *z*+1/2.
